# Supplementary material for: Uncovering a novel function of the CCR4-NOT complex in phytochrome A-mediated light signalling in plants
Source: eLife. 2021 Mar 30;10:e63697. doi: 10.7554/eLife.63697 (PMC8009681; doi:10.7554/eLife.63697)
Supplement: Supplementary file 1 [file elife-63697-supp2.odt]

Supplementary File 2. Cloning of constructs and mutants/transgenic lines used in this study.

Supplementary File 2A. Plasmids used in this study and cloning of plasmid constructs.

| Name/number | Insert | Vector | Cloning strategy / reference |
| --- | --- | --- | --- |
| pBRIDGE | BD, Aux-HA |  | Takara Clontech, Kyote, Japan |
| pcDNA3.1 |  |  | Thermo Fisher Scientific |
| pGADT7 | AD |  | Takara Clontech, Kyoto, Japan |
| pGBKT7 | BD |  | Takara Clontech, Kyoto, Japan |
| pJET1.2 |  |  | Thermo Fisher Scientific |
| p35S:P19 | p35S:P19 |  | (Chapman et al., 2004) |
| 156_pFRETrv-2in1-NC |  |  | (Hecker et al., 2015) |
| #43 pCHF5 | p35S |  | Gift by C. Fankhauser (Hiltbrunner et al., 2005) |
| #253 pCHF20 | p35S:GFP |  | (Hiltbrunner et al., 2006) |
| #295 pCHF40 | p35S:CFP |  | (Hiltbrunner et al., 2005) |
| #401 pCHF70 | p35S:YFP |  | (Hiltbrunner et al., 2005) |
| #403 pCHF30 | p35S:YFP |  | (Hiltbrunner et al., 2006) |
| #830 pGADT7ah-FHY1 | AD-FHY1 | pGADT7ah | (Hiltbrunner et al., 2005) |
| #942 pphyA30-phyA | pPHYA:PHYA-YFP | pCHF | (Hiltbrunner et al., 2006) |
| #1079 D153 | BD |  | (Hiltbrunner et al., 2006) |
| #1123 pGBKT7ah-FHY1 | BD-FHY1 | pGBKT7ah | FHY1 CDS and flanking sequence was cut from #830 pGADT7-FHY1 (Hiltbrunner et al., 2005) using EcoRI and PstI, and ligated into pGBKT7 (Takara Clontech) opened with EcoRI and PstI. |
| #1239 D153ah-phyA | phyA-BD | D153ah | (Hiltbrunner et al., 2006) |
| #1419 D153ah- phyA 1-100 | phyA 1-100-BD | D153ah | (Hiltbrunner et al., 2006) |
| #1442 D153ah- phyA 1-406 | phyA 1-406-BD | D153ah | (Hiltbrunner et al., 2006) |
| #1445 D153ah- phyA 1-617 | phyA 1-617-BD | D153ah | (Hiltbrunner et al., 2006) |
| #1448 D153ah- phyA 563-1122 | phyA 563-1122-BD | D153ah | (Hiltbrunner et al., 2006) |
| #1454 pCHF5v1 | p35S | pCHF5 | (Sheerin et al., 2015) |
| #1503 D153ah- phyA 1-200 | phyA 1-200-BD | D153ah | PHYA 1-200 CDS was PCR amplified from #1239 D153ah-phyA (Hiltbrunner et al., 2006) using primers ah013 and ah270. The PCR fragment was cut with BamHI and SpeI and ligated into #1239 D153ah-phyA opened with BamHI and SpeI to replace full-length PHYA CDS. |
| #1506 D153ah- phyA 1-308 | phyA 1-308-BD | D153ah | PHYA 1-308 CDS was PCR amplified from #1239 D153ah-phyA (Hiltbrunner et al., 2006) using primers ah013 and ah269. The PCR fragment was cut with BamHI and SpeI and ligated into #1239 D153ah-phyA opened with BamHI and SpeI to replace full-length PHYA CDS. |
| #1509 D153ah- phyA 101-308 | phyA 101-308-BD | D153ah | PHYA 101-308 CDS was PCR amplified from #1239 D153ah-phyA (Hiltbrunner et al., 2006) using primers ah271 and ah269. The PCR fragment was cut with BamHI and SpeI and ligated into #1239 D153ah-phyA opened with BamHI and SpeI to replace full-length PHYA CDS. |
| #1511 D153ah- phyA 101-406 | phyA 101-406-BD | D153ah | PHYA 101-406 CDS was PCR amplified from #1239 D153ah-phyA (Hiltbrunner et al., 2006) using primers ah271 and ah219. The PCR fragment was cut with BamHI and SpeI and ligated into #1239 D153ah-phyA opened with BamHI and SpeI to replace full-length PHYA CDS. |
| #1515 D153ah- phyA 101-617 | phyA 101-617-BD | D153ah | PHYA 101-617 CDS was PCR amplified from #1239 D153ah-phyA (Hiltbrunner et al., 2006) using primers ah271 and ah203. The PCR fragment was cut with BamHI and SpeI and ligated into #1239 D153ah-phyA opened with BamHI and SpeI to replace full-length PHYA CDS. |
| #1535 pCHF90 | p35S:LUC |  | (Sheerin et al., 2015) |
| #1548 D153ah- phyA 1-406 Δ6-12 | phyA 1-406 Δ6-12-BD | D153ah | (Trupkin et al., 2007) |
| #1588 D153ah- phyA Δ6-12 | phyA Δ6-12-BD | D153ah | (Trupkin et al., 2007) |
| #1622 pCHF72 | p35S:NLS-YFP |  | (Genoud et al., 2008) |
| #1874 pPPO30 | p35S:YFP |  | (Rausenberger et al., 2011) |
| #1994 D153ah-phyA-NLS | PHYA-NLS-BD | D153ah | A fragment coding for the C-terminal part of phyA and an SV40 NLS was cut from #1995 D153ah-phyA Y242H-NLS (Rausenberger et al., 2011) using XbaI and SpeI, and ligated in sense orientation into #1239 D153ah-phyA (Hiltbrunner et al., 2006) cut with XbaI and SpeI. |
| #1995 D153ah-phyA Y242H-NLS | PHYA Y242H-NLS-BD | D153ah | (Rausenberger et al., 2011) |
| #2001 pPPO30-phyA-NLS | p35S:PHYA NLS-YFP | pPPO30 | A fragment coding for phyA-NLS was cut from #1994 D153ah-phyA-NLS using BamHI and SpeI, and ligated into #1874 pPPO30 (Rausenberger et al., 2011) digested with BamHI and XbaI. |
| #2095 pPPO30A-phyA-NLS | pPHYA:PHYA NLS-YFP | pPPO30A | A fragment coding for the PHYA promoter was cut from #942 pphyA30-phyA (Hiltbrunner et al., 2006) using StuI and BamHI, and ligated into #2001 pPPO30-phyA-NLS digested with PmeI and XbaI. |
| #2698 pCHF91 | p35S:LUC | pCHF5 | (Sheerin et al., 2015) |
| #2946 pCHF70HA | p35S:HA-YFP | pCHF5 | (Enderle et al., 2017) |
| #4865 pCHF230 | p35S:tagRFP | pCHF5 | tagRFP CDS was PCR amplified from 156_pFRETrv-2in1-NC (Hecker et al., 2015) using primers CM040 and CM041. The resulting PCR fragment was digested with BamHI and SpeI and ligated into pCHF5 (Hiltbrunner et al., 2005) opened with BamHI and XbaI. |
| #5062 pCHF230-phyA-NLS | p35S:PHYA-NLS-tagRFP | pCHF230 | PHYA-NLS CDS was cut from #1994 D153ah-phyA-NLS with BamHI and SpeI, and ligated into #4865 pCHF230 opened with BamHI and XbaI. |
| #6067 pSEL | p35S | pCHF5 | A fragment was PCR amplified from #1535 pCHF90 (Sheerin et al., 2015) using primers ah1473 and ah1474. The resulting PCR fragment was then used as template for another PCR with primers ah1473 and ah1475. The fragment resulting from this PCR was then used for a third PCR with primers ah1473 and ah1476. The resulting fragment was digested with PstI and XhoI and ligated it into #43 pCHF5 (Hiltbrunner et al., 2005) opened with SbfI and XhoI. |
| #6112 pSUL | p35S | pSEL | The CDS coding for Sulfadiazine resistance was amplified from pGGF012 (Lampropoulos et al., 2013) using primers ah1471 and ah1472. The resulting PCR fragment was ligated by Gibson assembly (Gibson et al., 2009) into #6067 pSEL opened with SnaBI and StuI. |
| #t63 | YFP-3×HA | pJET1.2 | EYFP CDS was PCR amplified from #403 pCHF30 (Hiltbrunner et al., 2006) using primers tx009 and tx010; tx010 includes the 3×HA tag. The resulting PCR fragment was used as template for another PCR with primers tx047 and tx048. The resulting PCR fragment was blunt-end ligated into pJET1.2 (Thermo Fisher Scientific). |
| pDS50 pGBKMCS | BD-MCS | pGBKT7 | (Sheerin et al., 2015) |
| pDS51 pGADMCS | AD-MCS | pGADT7 | (Sheerin et al., 2015) |
| pDS67 pBridgeDS3 | C-term-BD, Aux-HA | pBRIDGE | pBRIDGE (Takara Clontech) was digested with XbaI/PstI. The resulting 850 bp fragment and annealed oligos DS224/DS225 were ligated into pBRIDGE opened with HindIII and XbaI to replace the GAL4-BD with a new MCS. The GAL4 BD was then excised from pD153ah (Hiltbrunner et al., 2006) with SpeI/EcoRI and cloned into the new SpeI/EcoRI sites. Subsequently the new vector was digested with NotI/BglII and annealed oligos DS211/DS212 were inserted. Lastly a PCR fragment amplified from pBRIDGE with primers DS205/DS226 was cut with XbaI/PstI and ligated into the XbaI/PstI cut vector. |
| pDS361 pCHF70HA-NOT9B | p35S:HA-YFP-NOT9B | pCH70HA | NOT9B CDS (AT5G12980.1) was amplified from Col‑0 cDNA using DS160 and DS161. The resulting PCR product was digested using BamHI and XbaI, and ligated into #2946 pCHF70HA (Enderle et al., 2017) opened with BamHI and XbaI. |
| pDS376 | pNOT9B | pCHF5v1 | #1454 pCHF5v1 (Sheerin et al., 2015) was cut with HindIII, blunted with Klenow, and then cut with SalI to obtain a ~660 bp SalI-HindIII fragment blunted at the HindIII site. This fragment was ligated together with annealed oligos DS356/DS357 into #1454 pCHF5v1 opened with NcoI and SmaI. The resulting vector was then opened with NcoI and XmaI and ligated with the NOT9B promoter (pNOT9B) digested with NcoI and XmaI. pNOT9B was PCR amplified from Col‑0 genomic DNA using the primers DS295 and DS296. |
| pDS396 | pNOT9B:GUS | pCHF5v1 | GUS CDS was PCR amplified from pPCH1:GUS (Enderle et al., 2017) using primers ah396 and ah397. The PCR fragment was digested with BglII and ligated in sense orientation into pDS376, which was opened with BamHI and dephosporylated. Finally, for cloning of other constructs derived from this vector, the NheI site in the vector backbone was deleted as described for pPPOv3 (Enderle et al., 2017). |
| pDS398 | pNOT9:LUC-NOT9B | pCHF5v1 | Firefly LUC CDS was cut from #2698 pCHF91 (Sheerin et al., 2015) using XmaI and BamHI and ligated into pDS376 opened with XmaI and BamHI. The resulting vector was then cut with BamHI and SpeI and ligated with NOT9B CDS cut from pDS361 using BamHI and XbaI. |
| pDS402 pGAD-NOT9B | AD-NOT9B | pGADMCS | NOT9B CDS (AT5G12980.1) was PCR amplified from Col‑0 cDNA using DS160 and DS161. The resulting PCR product was digested using BamHI and XbaI, and ligated into pDS51 pGADMCS opened with BamHI and SpeI. |
| pDS448 pBridgeDS3-PHYA | PHYA-BD, Aux-HA | pDS67 | The PHYA CDS was cut from #1239 pD153ah-PHYA (Hiltbrunner et al., 2006) with BamHI/SpeI and ligated into BamHI/SpeI cut pDS67. |
| pDS484 pGAD-NOT9A | AD-NOT9A | pGADMCS | NOT9A CDS (AT3G20800.1) was PCR amplified from Col‑0 cDNA using DS307 and DS308. The resulting PCR product was digested using BglII and XbaI, and ligated into pDS51 pGADMCS opened with BamHI and SpeI. |
| pDS485 pGAD-NOT9C | AD-NOT9C | pGADMCS | NOT9C CDS (AT2G32550.2) was PCR amplified from Col‑0 cDNA using DS305 and DS306. The resulting PCR product was digested using BglII and XbaI, and ligated into pDS51 pGADMCS opened with BamHI and SpeI. |
| pDS700 pcDNA3.1-mCherry | FLAG-myc-mCherry | pcDNA3.1 | (Enderle et al., 2017) |
| pMB04 pGAD-NOT9B ΔGWB-LUC | AD-NOT9B ΔGWB-LUC = AD-NOT9B R244E A248L R205D H208D-LUC | pPS137 | AD-NOT9B R244E A248L R205D H208D-LUC CDS was generated by amplifying NOT9B-LUC from pPS137 in three fragments with overlaps containing point mutations. They were generated by PCR on pPS137 using primers oMB06 and oPS245 (fragment 1), oMB03 and oMB04 (fragment 2), and oMB05 and oPS242 (fragment 3). All three PCR fragments were ligated by Gibson assembly (Gibson et al., 2009) into pPS137 opened with BamHI and SpeI. |
| pMB06 pGAD-NOT9B-ΔPNB-LUC | AD-NOT9B ΔPNB-LUC = AD-NOT9B H58A F60A A64Y V71Y-LUC | pPS137 | AD-NOT9B H58A F60A A64Y V71Y-LUC CDS was generated by amplifying NOT9B-LUC from pPS137 in two fragments with overlaps containing point mutations. They were generated by PCR on pPS137 using primers oMB15 and oPS242 (fragment 1), and oPS245 and oMB16 (fragment 2). The two PCR fragments were ligated by Gibson assembly (Gibson et al., 2009) into pPS137 opened with BamHI and SpeI. |
| pMBag06 pCHF70HA-NOT9B ΔPNB | p35S:HA-YFP-NOT9B ΔPNB = p35S:HA-YFP-NOT9B H58A F60A A64Y V71Y | pCHF70HA | NOT9B H58A F60A A64Y V71Y CDS was amplified from pMB06 using oMBag01 and oMBag02. The resulting fragment was ligated by Gibson assembly (Gibson et al., 2009) into #2946 pCHF70HA (Enderle et al., 2017) opened with BamHI and XbaI. |
| pPS01 | NOT1 | pJET1.2 | NOT1 CDS (AT1G02080.1) was ordered as gBlocks aPS01, aPS02, aPS03, and aPS04 (IDT) with 30 bp overlaps to each other and pJET1.2 (Thermo Fisher Scientific). A BamHI site was included at the 5’ end of NOT1 (in aPS01) and an NheI site at the 3’ end (in aPS04). The four gBlocks were ligated into pJET1.2 by Gibson assembly (Gibson et al., 2009). |
| pPS02 | NOT2B | pJET1.2 | NOT2B CDS (AT5G59710.1) was ordered as gBlock aPS05 (IDT) including a BamHI site at the 5’ end and an NheI site at the 3’ end, and blunt-end ligated into pJET1.2 (Thermo Fisher Scientific). |
| pPS03 | CCR4A | pJET1.2 | CCR4A CDS (AT3G58560.1) was ordered as gBlock aPS06 (IDT) including a BamHI site at the 5’ end and an NheI site at the 3’ end, and blunt-end ligated into pJET1.2 (Thermo Fisher Scientific). |
| pPS04 | NOT4B | pJET1.2 | NOT4B CDS (AT3G45630.1) was ordered as gBlocks aPS09 and aPS10 (IDT) with 30 bp overlaps to each other and pJET1.2 (Thermo Fisher Scientific), and including a BamHI site at the 5’ end (in aPS09) and an NheI site at the 3’ end (in aPS10). The two gBlocks were ligated into pJET1.2 by Gibson assembly (Gibson et al., 2009). |
| pPS05 | NOT4A | pJET1.2 | NOT4A CDS (AT5G60170.2) was ordered as gBlocks aPS07 and aPS08 (IDT) with 30 bp overlaps to each other and pJET1.2 (Thermo Fisher Scientific), and including a BamHI site at the 5’ end (in aPS07) and an NheI site at the 3’ end (in aPS08). The two gBlocks were ligated into pJET1.2 by Gibson assembly (Gibson et al., 2009). |
| pPS06 | CAF1A | pJET1.2 | CAF1A CDS (AT3G44260.1) was amplified from Col‑0 cDNA using primers oPS07 and oPS08, including a BamHI and an NheI site, respectively. The PCR product was blunt-end ligated into pJET1.2 (Thermo Fisher Scientific). |
| pPS07 | CAF1B | pJET1.2 | CAF1B CDS (AT5G22250.1) was amplified from Col‑0 cDNA using primers oPS05 and oPS06, including a BamHI and an NheI site, respectively. The PCR product was blunt-end ligated into pJET1.2 (Thermo Fisher Scientific). |
| pPS08 pGADT7ah-NOT1 | AD-NOT1 | pGADT7ah | NOT1 CDS (AT1G02080.1) was cut from pPS01 with BamHI and NheI, and ligated into #830 pGADT7-FHY1 (Hiltbrunner et al., 2005) opened with BamHI and SpeI to replace FHY1. |
| pPS09 pGBKT7ah-NOT1 | BD-NOT1 | pGBKT7ah | NOT1 CDS (AT1G02080.1) was cut from pPS01 with BamHI and NheI, and ligated into #1123 pGBKT7ah-FHY1 opened with BamHI and SpeI to replace FHY1. |
| pPS10 pGADT7ah-CCR4A | AD-CCR4A | pGADT7ah | CCR4A CDS (AT3G58560.1) was cut from pPS03 with BamHI and NheI, and ligated into #830 pGADT7-FHY1 (Hiltbrunner et al., 2005) opened with BamHI and SpeI to replace FHY1. |
| pPS11 pGBKT7ah-CCR4A | BD-CCR4A | pGBKT7ah | CCR4A CDS (AT3G58560.1) was cut from pPS03 with BamHI and NheI, and ligated into #1123 pGBKT7ah-FHY1 opened with BamHI and SpeI to replace FHY1. |
| pPS12 pGADT7ah-CAF1A | AD-CAF1A | pGADT7ah | CAF1A CDS (AT3G44260.1) was cut from pPS06 with BamHI and NheI, and ligated into #830 pGADT7-FHY1 (Hiltbrunner et al., 2005) opened with BamHI and SpeI to replace FHY1. |
| pPS13 pGBKT7ah-CAF1A | BD-CAF1A | pGBKT7ah | CAF1A CDS (AT3G44260.1) was cut from pPS06 with BamHI and NheI, and ligated into #1123 pGBKT7ah-FHY1 opened with BamHI and SpeI to replace FHY1. |
| pPS14 pGADT7ah-CAF1B | AD-CAF1B | pGADT7ah | CAF1B CDS (AT5G22250.1) was cut from pPS07 with BamHI and NheI, and ligated into #830 pGADT7-FHY1 (Hiltbrunner et al., 2005) opened with BamHI and SpeI to replace FHY1. |
| pPS15 pGBKT7ah-CAF1B | BD-CAF1B | pGBKT7ah | CAF1B CDS (AT5G22250.1) was cut from pPS07 with BamHI and NheI, and ligated into #1123 pGBKT7ah-FHY1 opened with BamHI and SpeI to replace FHY1. |
| pPS16 pGADT7ah-NOT4A | AD-NOT4A | pGADT7ah | NOT4A CDS (AT5G60170.2) was cut from pPS05 with BamHI and NheI, and ligated into #830 pGADT7-FHY1 (Hiltbrunner et al., 2005) opened with BamHI and SpeI to replace FHY1. |
| pPS17 pGBKT7ah-NOT4A | BD-NOT4A | pGBKT7ah | NOT4A CDS (AT5G60170.2) was cut from pPS05 with BamHI and NheI, and ligated into #1123 pGBKT7ah-FHY1 opened with BamHI and SpeI to replace FHY1. |
| pPS18 pGADT7ah-NOT4B | AD-NOT4B | pGADT7ah | NOT4B CDS (AT3G45630.1) was cut from pPS04 with BamHI and NheI, and ligated into #830 pGADT7-FHY1 (Hiltbrunner et al., 2005) opened with BamHI and SpeI to replace FHY1. |
| pPS19 pGBKT7ah-NOT4B | BD-NOT4B | pGBKT7ah | NOT4B CDS (AT3G45630.1) was cut from pPS04 with BamHI and NheI, and ligated into #1123 pGBKT7ah-FHY1 opened with BamHI and SpeI to replace FHY1. |
| pPS20 pGADT7ah-NOT9B | AD-NOT2B | pGADT7ah | NOT2B CDS (AT5G59710.1) was cut from pPS02 with BamHI and NheI, and ligated into #830 pGADT7-FHY1 (Hiltbrunner et al., 2005) opened with BamHI and SpeI to replace FHY1. |
| pPS21 pGBKT7ah-NOT9B | BD-NOT2B | pGBKT7ah | NOT2B CDS (AT5G59710.1) was cut from pPS02 with BamHI and NheI, and ligated into #1123 pGBKT7ah-FHY1 opened with BamHI and SpeI to replace FHY1. |
| pPS30 pCHF70-NOT9C | p35S:YFP-NOT9C | pCHF70 | NOT9C CDS (AT2G32550.2) was amplified from Col‑0 cDNA using oPS11 and oPS12, and blunt-end ligated into pJET1.2 (Thermo Fisher Scientific). The resulting plasmid was cut with BglII and AvrII, and the insert ligated into #401 pCHF70 (Hiltbrunner et al., 2005) opened with BamHI and XbaI. |
| pPS31 pCHF70-NOT9A | p35S:YFP-NOT9A | pCHF70 | NOT9A CDS (AT3G20800.1) was amplified using oPS13 and oPS14, and blunt-end ligated into pJET1.2 (Thermo Fisher Scientific). The resulting plasmid was cut with BglII and NheI, and the insert ligated into #401 pCHF70 (Hiltbrunner et al., 2005) opened with BamHI and XbaI. |
| pPS74 pCHF40-DCP1 | p35S:DCP1-CFP | pCH40 | DCP1 CDS (AT1G08370.1) was amplified from Col‑0 cDNA using primers oPS110 and oPS111. The resulting fragment was digested with BamHI and SpeI, and ligated into #295 pCHF40 (Hiltbrunner et al., 2005) opened with BamHI and XbaI. |
| pPS95 | GFP | pcDNA3.1 | GFP CDS was PCR amplified with oPS118 and oPS119 from pcDNA3.1-g2mGFP; PHYA CDS was amplified from #1239 D153ah-phyA with oPS120 and oPS121. Both fragments were ligated by Gibson assembly (Gibson et al., 2009) into pcDNA3.1 opened with BamHI and Acc65I. |
| pPS101 | FLAG-myc-mCherry-NOT9B | pcDNA3.1 | NOT9B CDS was PCR amplified from pDS402 using oPS126 and oPS127. mCherry CDS was amplified from DS700 using oPS125 and oPS124. Both fragments were ligated by Gibson assembly (Gibson et al., 2009) into pcDNA3.1 opened with BamHI and Acc65I. |
| pPS104 | GFP | pcDNA3.1 | (Enderle et al., 2017) |
| pPS106 pGBKT7ah-AGO1 | BD-AGO1 | pGBKT7ah | AGO1 CDS (AT1G48410.2) was amplified by PCR from Col‑0 cDNA using oPS136 and oPS137. The resulting PCR fragment was digested with BglII and SpeI and ligated into #1123 pGBKT7ah-FHY1 opened with BamHI and SpeI to replace FHY1. |
| pPS137 pGADT7ah-NOT9B-LUC | AD-NOT9B-LUC | pGADT7 | NOT9B CDS (AT5G12980.1) was amplified by PCR from pDS402 pGAD-NOT9B using oPS241 and oPS242. Firefly LUC CDS was amplified from pDS398 using oPS243 and oPS244. The two fragments were ligated by Gibson assembly (Gibson et al., 2009) into #830 pGADT7-FHY1 (Hiltbrunner et al., 2005) opened with BamHI and SpeI to replace FHY1. |
| pPS166 pCHF72-NOT9B | p35S:NLS-YFP-NOT9B | pCHF72 | NOT9B CDS (AT5G12980.1) was cut from pDS361 using BamHI and SalI and ligated into #1622 pCHF72 (Genoud et al., 2008) opened with BamHI and SalI |
| pPS169 pCHF70HA-NOT9B ΔGWB | p35S:HA-YFP-NOT9B ΔGWB = p35S:HA-YFP-NOT9B R244E A248L R205D H208D | pCHF70HA | NOT9B R256E A260L R217D A220D CDS was amplified from pMB04 using oMBag01 and oMBag02. The resulting PCR fragment was ligated by Gibson assembly (Gibson et al., 2009) into pCHF70HA (Enderle et al., 2017) opened with BamHI and XbaI. |
| pPS182 | p35S:FLAG-myc-mCherry | pCHF5 | FLAG-myc-mCherry CDS was amplified from pDS700 pcDNA3.1-mCherry using primers oPS324 and oPS325. The resulting PCR fragment was digested using BglII and SalI and ligated into pCHF5 (Hiltbrunner et al., 2005) opened with BamHI and SalI. |
| pPS183 | p35S:FLAG-myc-mCherry-NLS-NOT1 M | pPS182 | NOT1 M CDS was PCR amplified from pPS01 using primers oPS380 and oPS381. The resulting fragment was digested with XbaI and BamHI and ligated into pPS182 opened with BamHI and XbaI. |
| pPS187 | phyA-BD / HA-NOT1 MIF4G-GFP | pDS448 | NOT1 MIF4G CDS was amplified from pPS183 using oPS385 and oPS386. GFP CDS was PCR amplified from #253 pCHF20 (Hiltbrunner et al., 2006) using oPS387 and oPS388. The two fragments were ligated by Gibson assembly (Gibson et al., 2009) into pDS448 opened with NheI. |
| pPS188 | BD / HA-NOT1 MIF4G-GFP | pDS67 | NOT1 MIF4G CDS was amplified from pPS183 using oPS385 and oPS386. GFP was PCR amplified from #253 pCHF20 (Hiltbrunner et al., 2006) using oPS387 and oPS388. The two fragments were ligated by Gibson assembly (Gibson et al., 2009) into pDS67 opened with NheI. |
| pPS190 | BD- NOT1 MIF4G-GFP | pGBKMCS | NOT1 MIF4G-GFP CDS was cut from pPS187 using NheI and ligated in sense orientation into pDS50 pGBKMCS. pDS50 pGBKMCS was opened with SpeI and dephosphorylated. |
| pPS191 | p35S:FLAG-myc-mCherry-NLS-NOT1 M | pPS183 | Sulfadiazine resistance was cut from #6112 pSUL using XhoI and HindIII, and ligated into pPS183 opened with XhoI and HindIII to replace BASTA resistance. |
| pPS194 | p35S:FLAG-myc-mCherry | pPS182 | Sulfadiazine resistance was cut from#6112 pSUL using XhoI and HindIII, and ligated into pPS182 opened with XhoI and HindIII to replace BASTA resistance. |
| pPS197 | p35S:YFP-3×HA | pCHF5 | YFP-3×HA CDS was cut from #t63 using BamHI and SpeI, and ligated into #253 pCHF20 (Hiltbrunner et al., 2006) opened with BamHI and SpeI to replace GFP. |
| pPS198 | p35S:phyA-NLS-3×HA | pPS197 | PHYA-NLS CDS was excised from #1994 using BamHI and SpeI, and ligated into pPS197 opened with AvrII and BamHI. |
| pPS201 | p35S:FLAG-myc-mCherry-NLS- NOT1 MIF4G-mCherry | pPS182 | mCherry CDS was amplified from pPS183 using primers oPS418 and oPS419. NOT1 MIF4G CDS was amplified from pPS183 using primers oPS380 and oPS386. The two fragments were ligated by Gibson assembly (Gibson et al., 2009) into pPS183 opened with BamHI and XbaI to replace NOT1 M CDS. |
| pPS220 pCHF40-XRN4 | p35S:XRN4-CFP | pCHF40 | XRN4 CDS (AT1G54490.1) was amplified from Col‑0 cDNA using primers oPS170 and oPS171. The resulting PCR fragment was digested using BamHI and SpeI, and ligated into #295 pCHF40 (Hiltbrunner et al., 2005) opened with BamHI and SpeI. |
| pPS221 pCHF40-AGO1 | p35S:AGO1-CFP | pCHF40 | AGO1 CDS (AT1G48410.2) was amplified from pPS218 using primers oPS136 and oPS137. The resulting PCR fragment was digested using BamHI and SpeI, and ligated into #295 pCHF40 (Hiltbrunner et al., 2005) opened with BamHI and XbaI. |
| pPS227 | phyA-LUC-BD / Aux-HA | pDS448 | Firefly LUC CDS was PCR amplified from pDS398 using primers oPS444 and oPS445. The resulting fragment was cut with SpeI and ligated in sense orientation into pDS448. pDS448 was opened with SpeI and dephosphorylated. |
| pPS230 | phyA-LUC-BD / Aux-HA- NOT1 M-DUF | pDS448 | Firefly LUC CDS was PCR amplified from pDS398 using primers oPS444 and oPS445. The resulting fragment was cut with SpeI and ligated in sense orientation into pSD02. pSD02 was opened with SpeI and dephosphorylated. |
| pPS231 | LUC-BD / Aux-HA | pDS67 | Firefly LUC CDS was PCR amplified from pDS398 using primers oPS444 and oPS445. The resulting fragment was cut with SpeI and ligated in sense orientation into pDS67. pDS67 was opened with SpeI and dephosphorylated. |
| pPS232 | LUC-BD / Aux-HA-NOT1 M | pDS67 | Firefly LUC CDS was PCR amplified from pDS398 using primers oPS444 and oPS445. The resulting fragment was cut with SpeI and ligated in sense orientation into pSD06. pSD06 was opened with SpeI and dephosphorylated. |
| pPS245 pGBKT7ah-RH8 | BD-RH8 | pGBKT7ah | RH8 CDS (AT4G00660.1) was amplified from Col‑0 cDNA using primers oPS466 and oPS467. The resulting fragment was cut using BamHI and SpeI, and ligated into #1123 pGBKT7ah-FHY1 opened with BamHI and SpeI to replace FHY1. |
| pSD02 | phyA-BD / Aux-HA-NOT1 DUF | pDS448 | NOT1 DUF CDS was amplified from pPS01 using primers oSD03 and oSD04. The resulting fragment was digested with NheI and ligated in sense orientation into pDS448. pDS448 was opened with NheI and dephosphorylated. |
| pSD03 | phyA-BD / Aux-HA-NOT1 M | pDS448 | NOT1 M CDS was amplified from pPS01 using primers oSD05 and oSD06. The resulting fragment was digested with NheI and ligated in sense orientation into pDS448. pDS448 was opened with NheI and dephosphorylated. |
| pSD05 | BD / Aux-HA-NOT1 DUF | pDS67 | NOT1 DUF CDS was amplified from pPS01 using primers oSD03 and oSD04. The resulting fragment was digested with NheI and ligated in sense orientation into pDS67. pDS67 was opened with NheI and dephosphorylated. |
| pSD06 | BD / Aux-HA-NOT1 M | pDS67 | NOT1 M CDS was amplified from pPS01 using primers oSD05 and oSD06. The resulting fragment was digested with NheI and ligated in sense orientation into pDS67. pDS67 was opened with NheI and dephosphorylated. |
| pSD20 pGBK- NOT1 M | BD-NOT1 M | pGBKMCS | NOT1 M CDS was amplified from pPS01 using primers oSD05 and oSD06. The resulting PCR fragment was digested with NheI and ligated in sense orientation into pDS50 pGBKMCS. pDS50 pGBKMCS was opened with SpeI and dephosphorylated. |

Supplementary File 2B. Oligonucleotides used for cloning purposes.

| Name | Sequence (5’→3’) |
| --- | --- |
| ah013 | GGGGTACCGGATCCAAAAATGTCAGGCTCTAGGCCG |
| ah203 | GGACTAGTAGCTTCTAGTTCTTGTATACCA |
| ah219 | GGACTAGTCCCGGGGTTATCGAGTTCCACCTCCTTA |
| ah269 | GGACTAGTAAAGGAAAGCTTTTCATCTTG |
| ah270 | GGACTAGTTGATTGTAAGGCACCAGCAG |
| ah271 | CGCGGATCCAAAAATGGAGAATGCATCTGAGCTG |
| ah396 | GAAGATCTGATGTTACGTCCTGTAGAA |
| ah397 | GAAGATCTCCTGCAGGTTGTTTGCCTCCCTGCTG |
| ah1471 | TCGCATATCTCATTAAAGCAGGACTCTAGGATCGATCCCCCGGGTCACTAGGCATGATCTAACCCTC |
| ah1472 | AACAACATTACAATTACTATTTACAATTACCATGGCTTCTATGATATCCTCTTC |
| ah1473 | AAAACTGCAGGTCCTGCTGAGC |
| ah1474 | CATTACAATTACTATTTACAATTACGTAACTAGTAGGCCTAGAGTCCTGCTTTAATGAGA |
| ah1475 | AATTACCAACAACAACAAACAACAACAACATTACAATTACTATTTACAATTACGTAACTA |
| ah1476 | CCGCTCGAGTATAAGAGCTCTATTTTTACAACAATTACCAACAACAACAAACAACA |
| CM040 | CGCGGATCCCGGCTCTAGAATGGTGAGTAAAGGTGAAGAG |
| CM041 | GGACTAGTTTAGTTCAATTTGTGACCTAGCTTG |
| DS160 | ACGTCGGATCCAGGAATGGCTAATCTTCCTCCTCCT |
| DS161 | ACGTCTCTAGAGCCATTCACAAACATATGAGCCAACC |
| DS205 | ACGTCTCTAGAGCACATTCTGCG |
| DS211 | GGCCGGTAGATCTCTGCAGCCTAGGTAG |
| DS212 | GATCCTACCTAGGCTGCAGAGATCTACC |
| DS224 | AGCTGGATCCGGGCCCACTAGTGCAGAATTCTAATGCA |
| DS225 | TTAGAATTCTGCACTAGTGGGCCCGGATCC |
| DS226 | ACGTCCTGCAGTTAGCTAGCATGCAGATCTCCGGCCGCCACCTTTCTCTTCTTCTTAG |
| DS295 | ACGTCCCATGGATTCATTCCTTATATCTTTTTTCTGT |
| DS296 | ACGTCCCCGGGTAGAGCCCCGTCACCGTTTACACGCG |
| DS305 | ACGTCAGATCTAGGAATGTCGGAAAACATGGTGAATCT |
| DS306 | ACGTCTCTAGAGTTGTCCAGATTCTGAAGCA |
| DS307 | ACGTCAGATCTAGGAATGGCGAATCTACCTTCTTCT |
| DS308 | ACGTCTCTAGAAAGCATGTGCTCAAATCCTCCA |
| DS356 | CATGGTACCCGGGGCGGATCCAAAAACTAGTTAAG |
| DS357 | TCGACTTAACTAGTTTTTGGATCCGCCCCGGGTAC |
| oMBag01 | ACTCTCGGCATGGACGAGCTGTACAAGGCGGATCCGATGGCTAATCTTCCTCCTCCG |
| oMBag02 | CGATGATACGAACGAAAGCTCTGCAGGTCTCTAAGAATTCACAAACATATGAGCCAACCC |
| oMB03 | GACAGCGGAGGACTTCTTTGACCTGGGTCGGGTTTTGGGAAATATG |
| oMB04 | GAAGGCAACTTCCAAGTAGATCGCATGCTTCGGGGTTATCTGTCAAGCGAAGATAG |
| oMB05 | CTTGACAGATAACCCCGAAGCATGCGATCTACTTGGAAGTTGCCTTCCAGATCTTCTG |
| oMB06 | CCCAAAACCCGACCCAGGTCAAAGAAGTCCTCCGCTGTCGTGCACATGTACTC |
| oMB15 | GGTACCATTCCTTATCTTCTACAGGAGATTATCGCATATTACCCTGCCCTATCTCCTCCAACCATG |
| oMB16 | GATAATCTCCTGTAGAAGATAAGGAATGGTACCAGCAGAAGCCCATAAAAGCGGAGCCAAATCTTG |
| oPS05 | GATGGATCCCATGATCAAATCAGAAGCAGATCTCTC |
| oPS06 | GATGCTAGCAAAAACCTCTAAACCATACAAAACTC |
| oPS07 | GATGGATCCCATGGCGATCATTAAACCAAACCGAG |
| oPS08 | GATGCTAGCAAAAACCTCAAGCCCATACAAAACC |
| oPS11 | GATAGATCTCATGTCGGAAAACATGGTGAATCTACC |
| oPS12 | GATCCTAGGGTTGTCCAGATTCTGAAGCAGCTG |
| oPS13 | AGATCTCATGGCGAATCTACCTTCTTCTCTCTC |
| oPS14 | GATGCTAGCAAGCATGTGCTCAAATCCTCCACC |
| oPS110 | GATGGATCCATGTCTCAAAACGGGAAGATAATCCC |
| oPS111 | GATACTAGTTTGTTGAAGTGCATTTTGTAAAGTTCGG |
| oPS118 | CACTGCGGAACTCGCTGCAGCAAACAAGGGAACTAGTGGTGGCAGCGGAG |
| oPS119 | CAGAATTCCACCACACTGGACTAGTGGATCTTACTTGTACAGCTCGTCCATGCCGAG |
| oPS120 | CAAGCTGGCTAGCGTTTAAACTTAAGCTTGGATGTCAGGCTCTAGGCCGACTCAG |
| oPS121 | CTTGTTTGCTGCAGCGAGTTCCGC |
| oPS124 | CAAGCTGGCTAGCGTTTAAACTTAAGCTTGGATGGCCGAGGAGCAGAAGCTGATC |
| oPS125 | TCCGCCACTACCTCCAGATCCCTTGTACAGCTCGTCCATGCCGC |
| oPS126 | CTGTACAAGGGATCTGGAGGTAGTGGCGGAATGGCTAATCTTCCTCCTCCGCTCTC |
| oPS127 | CAGAATTCCACCACACTGGACTAGTGGATCTTAATTCACAAACATATGAGCCAACCCTTGAG |
| oPS136 | CTGATCTCAGAGGAGGACCTGCATATGGATCAAGATCTCATGGTGAGAAAGAGAAGAACGGATGCTC |
| oPS137 | GCCGCTGCAGGTCGACGGATCTTTAACTAGTGCAGTAGAACATGACACGCTTCACATTC |
| oPS170 | GCCGCTGCAGGTCGACGGATCTTTAACTAGTCAAGTTTGCACCTCGATGACTTGGTTG |
| oPS171 | CTGATCTCAGAGGAGGACCTGCATATGGATCCCATGGGAGTACCGGCGTTCTACAGATG |
| oPS241 | AAGCATACAATCAACTCCAAGCTTGGATCCATGGCTAATCTTCCTCCTCCGCTC |
| oPS242 | ATTCACAAACATATGAGCCAACCCTTGAG |
| oPS243 | CTCAAGGGTTGGCTCATATGTTTGTGAATGGTGGCAGCGGAGGCAGCATGGAAGACGCCAAAAACATAAAGAAAGGCC |
| oPS244 | CAGTAGCTTCATTTGCGGCCGCCGACTAGTCACGGCGATCTTTCCGCCCTTC |
| oPS245 | GTACCAGATTACGCTCATATGGATCCAGGAATGGCTAATCTTCCTCCTCCGCTC |
| oPS324 | CAGGTCGACTCATCTAGACCCGGGATCCGCC |
| oPS325 | CAGAGATCTATGGTAGACTACAAAGACGATGACGAC |
| oPS380 | CCGGCGGCATGGACGAGCTGTACAAGGCGGATCCCCCCGAGCCTCCAAAAAAGAAGAGAAAGGTCGAATTGGGAATGAGGGAAAACGCAATCGAGGCTC |
| oPS381 | CGAACGAAAGCTCTGCAGGTCGACTCATTCTAGAGTCACCAGCTGGATCGACTTGTG |
| oPS385 | GGTGGCGGCCGGAGATCTGCATGCTAGCATGAGGGAAAACGCAATCGAGGCTCC |
| oPS386 | TGGCGATGGAGTTGACTGGAACAGT |
| oPS387 | CAAGGACTGTTCCAGTCAACTCCATCGCCAGCTGGGAGCATGGTGAGCAAGGGCG |
| oPS388 | GATCCTACCTAGGCTGCAGTTAGCTAGCCTTGTACAGCTCGTCCATGCCGAG |
| oPS418 | CAAGGACTGTTCCAGTCAACTCCATCGCCAGCTGGGAGCATGGTGAGCAAGGGCGAGGAGGAT |
| oPS419 | CGAAAGCTCTGCAGGTCGACTCATCTAGACTTGTACAGCTCGTCCATGCCG |
| oPS444 | GATCACTAGTGGTGGCAGCGGAGGCAGC |
| oPS445 | GATCACTAGTCACGGCGATCTTTCCGCC |
| oPS466 | GAGGGATCCAATGAACAATCGAGGAAGGTACCCTCC |
| oSD03 | AGCGGCTAGCGTTTATACTAATACTTTGATGGATGAGGAG |
| oSD04 | AGCGGCTAGCAGCAGCGTCAGAGGATTCAGCCAG |
| oSD05 | AGCGGCTAGCAGGGAAAACGCAATCGAGGCTCC |
| oSD06 | AGCGGCTAGCGTCACCAGCTGGATCGACTTGTGAGCTCAAAAG |
| tx009 | CGCGGATCCAGCTAGCATGGTGAGCAAGGGCGAG |
| tx010 | CGGACTAGTTGCATAATCTGGAACATCGTATGGATACCCAGCATAATCAGGAACATCATAAGGGTATCCAGCGTAATCAGGCACATCATATGGATACCTAGGCTTGTACAGCTCGTCCATGC |
| tx047 | TTGGCGCGCCGGATCCAGCTAGCATGGTGAGCAAG |
| tx048 | TTGGCGCGCCTTAACTAGTTGCATAATCTGGAAC |

Supplementary File 2C. gBlocksTM used for cloning purposes. For the generation of plasmids coding for different putative CCR4-NOT complex subunits, gBlockTM fragments were ordered from IDT (Coralville, USA). Overlaps with pJET1.2 linear plasmid are in lowercase; coding sequence of respective genes in uppercase. Some of the fragment contain silent mutation that have been introduced for cloning reasons.

aPS01: NOT1 fragment 1

ggccgccagatcttccggatggctcgagtttttcagcaagatGGATCCCATGTATTCAAAGACAATTGAAAATCCAGACGAGACGAGTCCACGAATAATGTGGGCCTTAAAAAGAGTCAGCTACTCTCGTGGCTCCACCGAAAACCCTAGAAGGAAGAGCCCCTCTCCCGTCGTCCCTGGCATTTCGGATCTCGGATTTCCTTACCTCATGACTCCCTCCAAGGTCGCCGGTCATACCCGGTTCTTGCTCCACAGCTTTCATGATTCCGATGTCGACTCCATCGCCCTCCAGCTTTCTCAGTTTGTTGATTTTGGAGTCGAGACTAGTATTCCAGTTCTCAAGACCTGCCTGGATTGCTTCACCGCCCGGAGAAGCCATCCTAATTCGTTGCAGCTTGAGAAAGTTGTATCCTTGCTCTTTAAACATGTGCTCAAGTTGTCAAACCTTGCCACCCTGCTTCCTCACGCCCTCAATGATTTTGAGCTCACTCAAGAATCCGTGGATGATTTGACTACTACTTTGAATTTTTCAATCTCTGAGAATATTGGCTTTGCTCTCGCCCTGACCGATTTTGAAAGATTAGATGCAAAGACCACTGGACGGAACTTACTGTTGGCTCAGATTGAGCAACTCTGTGCCAATACTGGCCAGATTCTTTCATCTGAGCTAATCCATTCTGTTCTTTCGTTTCTCCGCAAATCTGAGGATCTTTCCATGCATTTGGATTCCTTCTTACAGTTTTTGTCTTCTGCTCAACCTAGAGATGACTTCTCTTTTGCTCTAACACCTATGCTTGCTCAACAAGTTCATGAAGCTCCTGTTTTCAGGAGCATGGATTTTCACACTGATTCTGCTGATAACGACCTTGATGCTATCTTAGCTGAAATCGATAAGGAAGTCAGCGTTGGAGATCTCATGGGAGAATTAGGTTGTGGATTCACTGCTGATGCCCAACAATGCAAAGAAATCTTGTCTAGTTTTGCTCCACTAGGAGAGGCTACTATCTCAAGAATTGTTGGCAATGTTTCTCGGACCTGTGCTGATCTTGAAGATAATCAAACCACCTTTTCAACGTTTACTGTAGCCCTTGGTTCCTGCATTCCAACTGAACTTCCCACGCCAAGGTCGTGGAATGTTGATATTCTAGTTGACACAATCAAGCAGCTTCAGGCTCCTGGCATAAGCTGGAGGAAAGTAATTGAGAATCTCGATCATGACGGATTTGACATCCCTAATATGGAGTCTTTCTCATTTTTTATGCGGATCTATAAAGCTGCTTGCAAGGAGCCATTTCCTCTTGATGCTGTATGTGGTTCTGTCTGGAAAAATATGGATGGTCAATTATCCTTTCTTAAGCACGCAATATCTGCTCCACCAGAAGTATTCACCTTTATGCATTCTCCAAGGAAGCTGGTATATATTGATAACATGCACAGCCAAGAGCAACAGTTAGGACTATCCAATCATGCATGGCTGTCGCTCGATCTCTTGGATGTACTGTGCCAATTGGCCGAGAGAGGTCATGCTGTTTTAGTTAGTTCGTTACTCCAGTATCCACTCACACAGTGTCCTAGAACCTTGCTTCTTGGAATGACACACATCAAAACTGCCTATAACCTCATACAGCGTGAAGTGGTTTCTGCTATTCTGCCAGTGATAATAACGAGTCCCCAGGATAGTGGTTTTATCCATAATCTCTGGCATCAAAATGCAGAACTCGTTCTGTGGGGGATTATAGATGCCCAACATCTCAAAGCAGACAGCATGCTCCGAATAATTGAAATCTGCCATGAGCT

aPS02: NOT1 fragment 2

GCAGACAGCATGCTCCGAATAATTGAAATCTGCCATGAGCTAAAGATTCTCTCTGTTGTCTTAGAATCAGTTCCAGTCTCATCCAGTATCAGATTGGCAGTCCTTGCCTCATTGAGAGGTCTTTTGGACATTGAGAACTGGTTGCCTAACTGCTTGTATATGTATAAGGATCTCTTTGCTGAGGAGTGCCTCAAATTTGTCAAAAATGTGCATTTTAGCGAATCAGACGATTTCAGGGCCAAAATTTTTCATCCTTCTGATCCATTATCAGATCTTCATTTGGAAGCAACAACTTCCCTTTTAAAAGTTTTGAAAGCTCATGATAATGCGATTACTTCATCTCAACTGGTCGAGGAGATTGAAAAAGTCAATGCAGCTATTTTGGATTGTAATCCTAAGCTGCAGAATGGTGAGGCTAAAGATTCATCAGCTCCCAATGCATATGGAGATGATGTTGAGGCAGAGGCAAACGCATATTTTCATCAAATGTTTTCTAGTCATTTGAGTGTTGACGCAATGGTTCAAATGCTTTCCCGATACAAGGAATCTTTGGTTCCAAGGGAAAAATTAATTTTTGAATGCATGATTGCTAATCTGTTTGAAGAATATCGGTTTTTCCCCAAGTATCCTGAGAGACAGCTGAAAATAGCCTCCATACTCTTTGGTTCTGTTATAAAACACCAGCTTATAAGTTCGCTCACTCTGGGGATGGCCCTGCGTCTAGTGTTGGATTCTTTGCGTAAACCTGCAGATTCAAAAATGTTTCTGTTTGGAAGCAAAGCTCTGGAACAGTTTGTGAATCGTCTGGTTGAATTGCCTCAATACTGTAACCATATTTTGCAAATATCTCATCTGCGTAGTACTCACCCGGAGTTAGTCACTGTTATTGAACAAGCACTTTCAAGGATTTCATCTGGTAATTTAGAATCAGATGCCTCTGTTTCACACCCTGGTCCCTCACAATCTTTCCCTGGAAATGGCGAGTTGAGTGGTTCTGGAATTGGCCAACCTGCATTACAGCTTTCTTCTCCCCTACAGCTTCAACAGAAAAATGAAGTGCCAAGTGTGCCATCTAACGAGGCAAAGCCACTTCTGCCATCGTTATCAACTACTTCCGTTGATGTTTCTGTCAATCCTAAGAATCCTGGCATTCCAACTTCTTCATCAACCTCAACTGGTTTTGTTCGTCCAGCTCGTGCAACCACTTCAACAAGGTTTGGCTCGGCTTTGAACATAGAAACCTTGGTTGCAGCAGCAGAAAGAAGGGAAAACGCAATCGAGGCTCCACCTTCAGATGTTCAGGACAAAGTTTCGTTCATAATCAATAATATCTCTACAACAAACATTGAATCTAAAGGGAAAGAGTTTGCTGAAATTTTGCCCCAACAGTATTATCCTTGGTTTGCACAGTACATGGTTATGAAAAGAGCGAGCATCGAGCCGAATTTTCATGATCTGTACCTGAAGTTCTTGGACAAAGTTGATTCCAAGCTGCTGTTTAAGGAGATACTTCAAAATACTTACGAAAATTGCAAGGTTCTTTTGGGCTCAGAGCTCATAAAGTCGAGTTCTGAAGAGCGTTCTCTTCTTAAAAATTTAGGCAGTTGGCTTGGAAGGTTGACCATTGGAAGGAATTATGTTTTAAGGGCGCGAGAAATAGATCCAAAATCCTTGATTGTGGAGGCCTATGAAAAAGGGTTGATGATAGCAGTCATTCCGTTCACGTCGAAAGTGCTGGAGCCATGCCAAAACAGTATTGCTTATCAACCTCCCAATCCTTGGACGATGGCTATACTAGGGTTGCTTGCTGAGATCTATTCAATGCCAAACCTAAAAATGAATCTGAAGTTTGACATAGAGGTTTTATTCAAGAACCTTGGTGTTGAAATGAAGGAAGTAGTGCCAACTTCCCTTCTAAAGGATCGGAAGAGAGAAATAGATGGCAATCCTGATTTTAGTAACAAAGATCCTGGAGTAACACAGATATCTCAGCCACA

aPS03: NOT1 fragment 3

AGTAACAAAGATCCTGGAGTAACACAGATATCTCAGCCACAGATGATTCCAGAGCCTAAAACAATTTCTCCTCTTAAACAAATTGACCTACCTCTTGATGTTGCAAATTCGCCTAATACAGATGTTCCCTCGAAGTTATTATCACAGTATGTAGCCCCACAACGTGTTTATACTAATACTTTGATGGATGAGGAGAAAGTAGCAACTCTTGGCTTGCCAGAACAGCTCCCGTCACCTCAAGGACTGTTCCAGTCAACTCCATCGCCATTGTTTTCTATTAGTCAGCTGTCAGCAGCACTTCCCAATATTGGAAATCATGTTGTTATCAATCAGAAATTAAGCGCATTTGGTATGCACTTTCCATTTCAAAGAGTGGTACCACTTGCCATGGACAGAGCTATCAAGGAGATTGTGTCTGGTATTGTTCAGCGAAGTGTTTGTATTGCGTGCCAAACAACAAAGGAGCTTGTGCTAAAGGATTACGCCCTGGAACCCGATGAGTCACGTATTTATAATGCAGCTCACTTGATGGTTGCGAGTTTAGCTGGGAGTTTGGCTCATGTGACTTGCAAGGAACCCCTGCGCACTTCAATATCCGGTCATCTACGGAATTCGCTTCAGGGTCTGAATATTTCAAATGATGCTCTTGAACAAATTGTGCAACTTGTCACCAATGACAACCTGGATTTGGGTTGTGCTGCTATTGAACAGGCTGCTACAGAAAAGGCAATACAAACAATTGATGCGGACATTGCTCAACAATTGTTGTTACGGAGAAAGCATAGAGATGGTGCTGGcTCCTCCTTTTTCGATCCAAACATTCTATCACAGAATTCCGTCAGTTTTATACCCGAATCCCTCCGTCCAAAACCTGGACACCTCTCCCTGTCTCAGCAGAGAGTTTATGAGGACTTTGTTCAACATCCTTGGCAAAAGCAGTCGACCCAAACTTCACACGGTTTATCTGCTGCATCAAGCTCATCTGGTGATGTTGCGCTTGGTAGTGGTTATGGTCCAGTATCAGGAAAAGTTGCTTCTGAATTTTTGTCTAATGCTGGAAACGCTCGGATGGACATGGTCTCCCGGCCATCAGATATTTCTGTGGATGGTTTTGAATCTTCTCCAGTCTCACTTTTGAGCTCACAAGTCGATCCAGCTGGTGACTCATCCAGTCTTCAATTTACTAAGTCACTACCAACCTCTGAATTGAATCTGGCTGAATCCTCTGACGCTGCTACGAAAGAAACTGGAACATCACTGCAGACATTGACTTCAGCTGCTACCATGGAACGACTTGGTGCTAGTAATATTACACAGCCTTCACTGTCCACGAGAGATGCACTGGATAAATGTCAAATTGTTACTCAGAAGATGGAGGAATTAGTGGCTAATAATGCAGGAGATGATGAAATCCAGGCCGTAATTTCTGAAGTTCCTGAAATCATCCTCAGATGTATAAGCAGAGATGAAGCCGCTTTTGCTGTCGCTCAAAAAGCTTTCAAGGCTCTTTACGAGAATGCATCAAGTAACCTTCACGTCAGTGCTAACCTAGCAATACTTGTTGCTATCCGTGATGTTTGCAAGCGTGTTGTTAAAGAGCTCACTAGTTGGGTGATTTATTCAGAGGAGGATCGGAAGCTCAACAAAGATATTACTATTGGTCTTATCCAGCGTGAGTTGCTTAGCCTAGCGGAGTACAATGTCCACATGGCG

aPS04: NOT1 fragment 4

TTAGCCTAGCGGAGTACAATGTCCACATGGCGAAGCATCTTGATGGAGGGAGAAACAAGACCGCAACTGACTTTGCTATTTCTCTACTCCAATCCTTGGTCACTGAGGAGTCGAGTGTCATTTCAGAGCTCCACAGTCTTGTTGATGCGCTGGCAAAGCTTGCCTCAAAATCCGGATCTTCTGAGTCATTGCAACAGCTAATTGACATCATACGAAATCCAGTTACTAACACAGCTGGCCTCTCTGATAGTTCAACCGGAAATGATAACAACGATAGGCAAAAGGATGAGAAGGTTGCGTGCAACACCACTAACACAGAAGAGAGTACCAGCTTGGATTATGTGGAATCAGATCCTGCGGGTTTCCAGAATCGGGTGTCCACGCTATTTAAAAACTGGTATCAAATCTGCGAACTTCCTGGTGCAAATGAAACCGCATGTTCACAATATGTCCTGCATTTGCACCAGACTGGATTACTTAAGGGAGATGATACAACAGAGAGTTTTTTCCGAATTCTTCTGGAACTTTCTGTTGCTCATTGTATATCTTCTGAAGATATTAATTCCGGTGCTGTGCAATCTCCTCAGCAACCTCAGAGTCCATCATTCCTTATCATTGATATGTATGCAAAGCTTGTTTTTTCAATATTGAAGTATTTCCCTGAGCAGGAGTCTAGCAGTAGGTTATTTCTTCTTTCAGAGATCATGGCTGACACAGTGAGATTTATTCAAAAGGACGCAGAAGATAAAAAGACATCACTCAATTCTAAACCATATTTCCGATTGTTCATCAACTGGCTACTGGACTTGTGTTCCCTGGAcCCTGGGACGGATGGTGCAAACTTTCAGGTTCTCACAGCTTTTGCCAACGCCTTCCACGCATTGCAGCCTCTTAAGATTCCTGCTTTCAGTTTTGCATGGCTAGAACTTGTCAGCCACAGAAGCTTCATGCCCAAACTACTTACAGTAAATGGCCAGAAGGGTTGGCCATATGTTCAGCGCCTGCTGGTGGACTTGCTTCAGTTTCTTGAGCCATTTTTAAGAAATGCTGAACTCGGAGGACCGGTTCATTTCCTATACAAAGGGACATTGAGAGTATTACTGGTTCTGCTTCATGATTTCCCAGAGTTCCTATGTGATTATCATTTTACTTTCTGTGATGTGATTCCTTCAAGTTGCATACAAATGCGGAATATTATCCTCAGTTCTTTTCCACGGAACATGAGGCTTCCTGATCCTTCTACCCCAAATTTAAAGATTGATTTGTTACCTGAGATAGTAGAAGCTCCCTGTATCCTTTCTGAGGTTGATGCTGCGCTAAAGGCAAAGCAAATGAAGAATGACGTGGATGAGTATCTTACGTCGAGGCAGCAAAATTCAACTTTCCTAAGTGAGTTGAAGACAAAGTTACTTCTATCGTCCAGCGAGGCTAGTTCAGCTGGAACCCGTTACAGTGTACCATTGATCAACTCGCTTGTGCTATATACTGGAATGCAGGCTATTCAGCAGCTTCAGGCCGGTGAGACACAGGCTCAAAATGTTGTGGCCTTGCAGATGTTCAAGTATTTAAGTATGGAACTTGATACGGAAGGGCGGTATCTGTTCCTTAATGCAATCGCCAATCAGCTTCGATACCCCAACAACCACACTCATTACTTTTCTTTCATCATGCTCTATCTATTCTTCGAATCTGATCAGGAGATCATACAGGAGCAAATAACTAGAGTGCTGTTGGAACGGCTAATTGTGAACAGGCCACATCCATGGGGACTTCTAATCACATTCATTGAGCTAATAAAGAACCCAAGATATAGCTTCTGGAAACAAGCCTTCATCAGATGTGCGCCTGAGATTGAGAAGCTGTTTGAATCGGTAGCCAGATCCTGTGGTGGCCTGAAGCCTGTCGACGAGGGAATGGTTTCGGGAGGTTGGGTCTCAGACAATTCTCACGCTAGCatctttctagaagatctcctacaatattctcagctgccatgg

aPS05: NOT2B

AGCGGATCCCATGTCAAACCTTCATTCATCTCTCAATGGTTCAGCTTCGAATTTACCAGATGGTTCGGGCAGATCGTTTACTGCTTCTTATTCTGGACAATCTGGTGCACCGTCTCCCTCTTTTCATCATACTGGAAATCTTCAGGGACTTCATAATATTCATGGGAACTATAATGTCGGGAACATGCAAGGTACACTTACATCAAGAAACTCAAGTATGAATAGTATACCATCTGCCGGAGTTCAACAACCTAATGGGAGTTTTTCCAGTGGAAGATTTGCTTCAAATAATCTACCTGTTAATCTCTCTCAGTTGTCCCATGGTAGCTCACATGGGCATTCGGGAATCCCAAATAGAGGTCTTAATGTTGTTGGCAACCCTGGATTCAGTAGTAACGCTAATGGAGTTGGTGGTTCTATTCCTGGAATTCTCTCTACATCTGCAGGACTCAGTAATCGGAATAGTGTTCCAGGGATGGGAATATCCCAATTGTTGGGAAATTCAGGTCCTCGAATAACAAATTCAATGGGAAACATGGTTGGTGGAGGTAACTTGGGGAGGAATATTAGCTCTGGTGGATTGTCTATTCCTGGTCTGTCTTCACGACTAAATTTGGCAGCAAATAGCGGATCAGGATTAAATGTTCAGGGACAGAACCGAATGATGGGTGGAGTACTTCCTCAAGGATCTCAGGTCATGTCTATGCTGGGGAACTCCTATCATACTGGTGGTGGCCCGCTTTCGCAGAATCATGTTCAGTCAGTTAACAATATGATGCTCAGTGATCATCCTAACGACAGCTCTCTATTTGACATCAACAACGATTTTCCCCAGCTGACAAGTCGTCCTGGGTCTGCTGGTGGCACTCAAGGACATCTAGGCTCTTTGAGGAAACAAGGTTTAGGAGTTCCACTTGTCCAACAAAACCAGGAGTTCAGCATCCAAAATGAAGACTTTCCTGCCCTTCCTGGATATAAAGGTGGTAATTCTGAGTATCCTATGGATTTGCATCAAAAAGAACAACTGCATGACAATGCTATGTCAATGATGCACTCTCAAAACTTTTCTATGGGTAGATCTGGTGGTTTCAACTTGGGAGCAACATATTCATCACATCGTCCACAACAACAGCCACAACATACTTCATCTACTGGTGGGCTACAGGGTCTTGGCCTTAGACCTCTAAGCTCGCCTAATGCAGTTTCCAGTATTGGTTATGATCAGCTTATTCAGCAGTATCAGCAACATCAAAATCAATCCCAGTTCCCTGTGCAACAGATGTCATCAATCAACCAATTTAGAGATTCTGAGATGAAATCGACACAGTCAGAGGCAGATCCTTTTTGCTTGCTTGGCTTGTTAGACGTACTAAACAGGAGCAACCCTGAATTGACCTCACTTGCTCTTGGCATCGACTTGACGACGCTAGGATTGGATTTGAATTCAACTGGAAATCTCTACAAGACATTTGCGTCTCCTTGGACAAATGAACCGGCAAAGAGCGAGGTCGAGTTCACAGTACCAAATTGTTACTACGCCACAGAACCTCCGCCTCTAACTCGAGCTAGTTTCAAAAGGTTCTCCTACGAGTTATTGTTCTACACATTTTACAGTATGCCAAAAGATGAAGCACAGCTGTACGCAGCAGATGAACTTTACGAAAGAGGTTGGTTTTACCACAAGGAACTCAGAGTATGGTTCTTCAGAGTCGGGGAACCTTTAGTCAGGGCAGCTACATATGAAAGAGGAACATACGAATACCTTGATCCAAATTCGTTCAAAACAGTGAGAAAGGAACATTTTGTTATCAAGTACGAGCTTATGGAAAAGAGACCAAGCTTGCTGCAGCTTGCTAGCAGC

aPS06: CCR4A

GCAGGATCCCATGCTTAGCGTTATACGAGTGCATCTACCGTCGGAGATTCCTATTGTTGGCTGCGAACTTACACCTTACGTACTTCTCCGGCGGCCTGATAAGACCCCCTCCACCGATGATGTGCCTGAGTCAGCCCCGCTTGAAGGTCACTTCTTAAAGTACAGATGGTTTCGAGTACAGAGTGATAAGAAAGTTGCGATTTGTAGTGTGCATCCATCCGAGACGGCAACATTGCAGTGTCTGGGATGTCTAAAGTCTAAAGTTCCTGTTGCCAAAAGCTACCATTGCTCTACTAAGTGCTTTTCGGATGCGTGGCAACATCACCGTGTGTTGCATGAGCGTGCTGCCAGTGCTGCTACAGAAGGAAATGATGAAGAAGAGTTGCCCCGTTTGAATAGCTCAGGGTCTGGATCTGGTGTTCTCAGCACCAGTGTTAGTTTAACGAATGGGTCATCATCAGTTTATCCTTCAGCCATAACTCAAAAGACCGGCGCCGGAGGAGAAACACTTGTTGAGGTTGGACGCTCTAAGACATACACACCAATGGCTGATGATATTTGCCATGTTTTGAAGTTTGAATGTGTAGTAGTCAATGCAGAGACAAAACAGAATGTGGGCCTTTCTTGTACCATATTGACTTCCCGTGTTATCCCAGCTCCATCACCTTCTCCGCGTAGGCTGATCTCTATTAGTGGAACTGATGTTACTGGGCACCTGGATTCTAATGGGCGGCCTTTGTCTATGGGAACATTCACTGTGCTGTCCTACAATATATTGTCTGATACATATGCCAGCAGCGACATATACAGTTACTGCCCCACATGGGCTCTCGCTTGGACATACCGTAGACAGAATTTGTTACGGGAAATTGTCAAGTACCGTGCAGATATAGTTTGTCTACAAGAGGTACAGAATGATCATTTTGAGGAGTTTTTTCTTCCTGAGCTGGATAAACACGGATACCAAGGTCTTTTTAAGAGGAAAACAAATGAGGTTTTCATCGGTAATACAAACACAATTGATGGGTGCGCTACTTTCTTCAGAAGAGACAGGTTCTCACATGTAAAAAAATATGAGGTTGAGTTCAACAAGGCTGCACAGTCTTTGACTGAGGCTATTATCCCTGTTTCCCAGAAGAAAAATGCTTTGAATCGGTTGGTCAAGGATAATGTTGCTTTAATAGTTGTACTGGAAGCAAAATTCGGTAGCCAGGCTGCTGACAATCCTGGGAAGCGCCAGCTACTTTGTGTGGCTAACACGCATGTAAACGTTCCTCATGAATTGAAAGATGTAAAGCTGTGGCAGGTTCACACATTATTGAAGGGACTGGAGAAAATAGCTGCCAGTGCAGATATTCCAATGCTTGTGTGTGGAGATTTCAACACAGTACCTGCGAGTGCTCCTCATACACTTCTTGCTGTGGGAAAGGTCGATCCATTGCACCCAGATTTGATGGTTGATCCTCTTGGAATTTTACGTCCTCACAGCAAGCTTACTCATCAGCTGCCACTGGTTAGTGCCTACTCACAATTTGCAAAAATGGGAGGCAACGTTATTACTGAGCAGCAACGTAGAAGACTGGAcCCTGCATCAAGTGAACCGTTGTTTACCAACTGTACCAGGGACTTTATAGGCACACTTGATTACATATTTTACACAGCGGACACGCTAACAGTGGAATCGTTGTTGGAACTGTTGGATGAAGAGTCCTTGAGGAAGGACACAGCTCTTCCATCTCCAGAATGGTCTTCTGATCACATTGCACTTTTGGCTGAATTTCGCTGCATGCCCAGGGCCAGACGAAACAATATTTTAGCTAGCACC

aPS07: NOT4A fragment 1

ggccgccagatcttccggatggctcgagtttttcagcaagatGGATCCCATGAGTGACCATGGTGAGAAGACTTGTCCACTTTGTGCTGAAGAGATGGATCTGACTGATCAACAATTGAAGCCTTGCAAATGTGGTTATCAGATTTGTGTTTGGTGCTGGCATCACATAGTGGACATGGCAGAGAAAGATCAAATAGAAGGGCGTTGTCCAGCTTGTCGCACTCCATATGACAAAGAAAAGATTGTAGGGATGACTGTTAATTGTGACAGTCTGGCTTCTGAAGGCAATATGGAGCGCAAGAAGATCCAGAAGTCAAAATCAAAATCTTCTGAAGGGAGAAAGCAACAACTCACCAGTGTGCGAGTGATCCAGAGGAATCTTGTTTACATTGTTGGGTTGCCTCTTAATCTAGCCGATGAAGATCTACTCCAACATAAAGAATATTTTGGTCAGTATGGAAAAGTTCTAAAGGTTTCCATGTCCCGAACAGCATCTGGTGTCATCCAACAATTCCCAAACAATACATGCAGTGTATATATTACTTATGGAAAAGAGGAAGAGGCTGTTCGCTGCATTCAAGCTGTTCATGGGTTTATCTTGGATGGTAAACCATTGAAGGCATGTTTTGGGACAACCAAGTATTGTCATGCATGGCTGAGAAATGTGGCATGCGTCAATCCTGATTGTCTCTATTTGCACGAGGTTGGTTCTCAAGATGATAGCTTCACAAAAGATGAGATCATATCAGCCTATACAAGGAGTAGAGTTCAACAAATCACTGGAGCAACAAATATTCTGCAGCACCATTCAGGGAACATGCTACCTCCACCACTGGATGCTTATTGCAGTGACAGTTCTTCTGCAAAACCAATTATAAAGGTTCCTTCAACTAATGCAACTAGTGTTCCCAGGTATTCTCCGCCAAGTGGGAGTGGGAGCTCCAGCAGATCCACTGCCCTTCCTGCTGCAGCGTCATGGGGGACACATCAGTCTTTAGCAACTTCGGTCACCTCAAATGGATCTTCTGATATACAAAGATCAACATCAGTGAATGGAACCTTACCCTTTTCTGCTGTTGTTGCAAACGCAGCTCATGGGCCTGTATCCTCTAATGACATTCTTAAAAGACCATCTCGCAAAGAAGAAAGCCAAATAGTTATGGACAAAGTCAAACCTAGTGTGTTGAAGCCCTTGCAGCATAATGTTGTGGTTTCTGGGTCCGAGAGAATTACCGCACCCGATAGAGATCCTACTAGCAATCGTTTATCCAGTTCAGTAGACTCTGCCTACGGAGGCAGAGACATTGACCAGCCTTCAGCCTACAGTGGCAGAGACATTGACAAGCCTTCATCTACTGTTAGCTCGTTTGATGCTGCAAATGAAGCTGTAGAAGATGTCCCTACTGTAAGCAACTTGTTGGATGGTGTTGCATGCATGCGGATAACTATGAATTGTAGGGATGAACGTCCTGATATTACAATGGCGATTGGTAGTCAAAGTGATCAAGGTTCTATTAGACAACCTGGTTCTGAAGTATCAAAGTTGCCA

aPS08: NOT4A fragment 2

GGTTCTATTAGACAACCTGGTTCTGAAGTATCAAAGTTGCCAGATTTAGAGCAATGTAGGATAGATAGTTCTATAAACACTGATAAGAAAGCTATCTCGTTAGAGGATAGAATCCCCCGCACTAGGCCAGGGTGGGATTGGATATCAGATTTGCAATCGCAAATGCAAGGAAGCTCTAAATTGCAAGTGGAGGATATATCAACATTGGATAGCCAACGGCCTCACCCTGAAGAGGATATTATCCACTCGCGGTTGCTGTCTAATTTGTCAAGTTCCAGTTTGGATACAAATCATATGGCTTCTCGTTCTTCCCTACCTTGTGAAGTTAGGGGCAGTGATAGATTGCATCTTCCAAATGGATTTGGTGAGAAATCCATGTCCAGTGTGGAGCACTCTCTGTTCGCAAATGAAGGCAGGAACAAAGTCAACAATGCAGAGGATGCTATACTTTCAAACATTTTGTCACTCGATTTTGATCCATGGGATGAATCCTTGACTTCTCCCCACAATTTGGCAGAGTTACTAGGTGAAGTTGATCAGCGATCTAGTACTCTTAAACCTTCAAACTTCCTAAAGCAACATAACAACCAGTCTAGATTTTCTTTTGCACGGTATGAAGAATCTAGTAATCAAGCATATGACAGTGAGAACTATAGCATTTATGGGCAGTTGTCAAGAGATAAACCTATTCAGGAGTCTGCCATGAGCCGAGATATCTATCGAAATAACCTTGGGAGTGTAAATGGATTTGCTTCAAACTTCGCTGGTGGATTGGATAACTTTGCCGCTAGTCCTTTATTTTCTTCGCACAAGAATCCTGTTTCACGACCTCAAGTTTCTGCGCCTCCAGGATTTTCTGCTCCCAACAGGCTACCTCCTCCTGGCTTCTCGTCACATGAAAGAGTGGGCCTATCATCAGATACTACACTAGGAAATCGATTTCTTGACTCTACTTCATTGAGGAATGCATATCAAGTACCACCTCCAGTTGGTAATTCAAATGGTGCAAGTGATATCGACTTTGTGGAcCCTGCAATTCTAGCTGTTGGAAGAGGGATGGTAAATGCTGACTTGGACATGAGATCAGGATTCTCATCACAACTGAATTCTTTTGAAAACGAAACAGGACTCCATATGTTGAGACAGCAGTCTCTGTCTTCTGCACAACAAGTTAATGGGTTTCATCATGATCTCAGAAACTTATCTCCTTCACTTAACGACCCTTACGGATTTAGCTCAAGGCTTATGGATCAAACACAGGGAAGTAGCTTGTCTCCTTTCTCACAGCTCCCAAGACAACAACCATCTGCAAATTCAATCTTGTCCAATGGTCATCACTGGGATAAGTGGAATGAAGGGCAAAGTGTGAACAATATAGGCATGGCCGAGCTTCTTAGGAATGAACGGCTGGGTTTTAATGGGAGCTTATACAACAACGGATATGAAGAACCAAAATTCCGGATTCCAAGTCCCGGTGATGTGTATAACAGGACATATGGGATTGCTAGCatctttctagaagatctcctacaatattctcagctgccatgg

aPS09: NOT4B fragment 1

ggccgccagatcttccggatggctcgagtttttcagcaagatGGATCCCATGAGTGATTACGGTGAAAAGACTTGCCCTTTGTGTGCTGAGGAGATGGATTTGACTGACCAGCAATTGAAGCCTTGCAAATGTGGCTATCAGATTTGTGTTTGGTGCTGGCACCATATAATGGACATGGCAGAGAAAGATCAATCAGAAGGGCGCTGTCCAGCTTGCCGCACTCCATATGACAAAGAAAAGATTGTTGGGATGACTGTTGATCAGGAGAGACTGGCCTCTGAAGGCAACATGGATCGCAAAAAAATACAAAAGTCGAAACCGAAATCTTCTGATGGGAGAAAGCCACTAACAAGTGTCCGAGTGGTTCAAAGAAATCTTGTTTACATTGTTGGATTGCCCCTTAATCTAGCAGATGAAGATCTTCTCCAGCGTAAAGAATATTTTGGTCAGTATGGAAAGGTTCTAAAGGTTTCGATGTCCCGAACAGCAACAGGTCTCATCCAACAGTTTCCTAACAACACATGCAGTGTATACATCACATATGGCAAAGAGGAAGAGGCCATTCGATGTATTCAGTCTGTTCATGGGTTTATCTTGGATGGTAAAGCACTGAAGGCATGTTTTGGGACAACCAAGTATTGCCATGCATGGTTGAGAAACGTGGCATGCAACAATCAGGATTGCCTCTATTTGCATGAGGTTGGCTCTCAAGAGGATAGTTTTACAAAAGATGAGATCATATCAGCCCATACAAGAGTTCAGCAAATCACTGGAGCAACAAATACTATGCAATACCGTTCAGGGAGCATGCTACCTCCACCATTGGATGCTTATACCAGTGACAGTTCTACTGGAAATCCAATTGCAAAAGTTCCTTCTAGTACTTCAGTTAGTGCTCCTAAGAGTTCCCCACCTAGTGGGAGCTCGGGTAAATCAACTGCTCTTCCTGCTGCAGCATCATGGGGGGCACGTTTAACAAATCAACACTCTTTAGCAACTTCAGCTTTGTCAAATGGATCTTTGGATAATCAAAGATCAACATCAGAGAATGGAACTTTGGCTACGTCTACAGTTGTTACAAAGGCAGCTAATGGGCCTGTATCCTCTAGTAACAGTCTTCAGAAAGCACCTCTCAAGGAAGAAATTCAATCATTGGCTGAAAAAAGCAAACCTGGTGTGTTGAAGCCTTTGCAGCAGAAGATTGTGTTGGAcCCCGAGTCCAAGAGAACTACATCACCTAATAGAGACCCTAGTAGCAATCAGATATCCTGTTTAGTAGAGTCTTCCTACAATAGCAGAGTCATTGACAAGCCTTCTGCTGTTGAGAATTCTCTTGAACACACAAGTGAAATTGCTGAAGATGTCTTCGATGTTGGTAAATTGTCTGCTGATGTTGCATGGATGGGGATAACTACAAATTCTAGGGATGAAACCCCTGGTGTTCCAGTGGTTATCGGTACTCATTGTGATCTTGGTTCTATTACACAATCTGATAACGATGTACAAAATCTCGAGCAATGTAGAAAACAGTCTCCTACAAACACTT

aPS10: NOT4B fragment 2

GAGCAATGTAGAAAACAGTCTCCTACAAACACTTATGCAGAAGCTGACATATCACTGAATGGAATTCATGGTTCAAGGCCTGAGTGGGATTGGAGATCAGGTTTGCAATCCCAGATAGATGTTAAGGAACCGTTGGAAGTCAATGATTTCTCATCATTCAATAACAACAGACGTGGTATTGCAGAGGCTGTTAGCCACTCGACTTCTAAGTTTAGTTCATCAATCTCAATTCTGGATTCAAATCATCTAGCTTCTCGTTCGTTCCAGAACCGGGAAACTTCTTGTGGGATGGATTCAAAAACTGGcTCCTCCTTTGAAATAGGTAGTGATAGATTGCATCTTCCAAATGGATTTTCTGAGAAAGCTATGTCCAATATGGAGCATTCATTATTTGCTAATGAAGGCAGAAGCAACATTCAGAATACAGAGGATGATATCATTTCAAACATTTTAGACTTTGATCCCTGGGATGAATCGTTGACTTCGCAACACAATTTTGCAAAATTACTTGGTCAGAGTGATCATAGGGCCAGTACTCTGGAATCTTCTAACCTCCTAAAGCAACATAATGACCAGTCTAGGTTCTCTTTTGCTCGGCATGAAGAATCAAATAGTCAAGCGTATGACAATAGAAGTTACAGCATATATGGGCAGTTGTCAAGAGATCAGCCTCTTCAAGAGTTTGGAGCGAACCGTGATATGTATCAGGATAAGCTTGGCAGTCAAAATGGTTTTGCCTCAAACTATTCCGGTGGATATGAACAATTTGCTACTAGTCCTGGATTATCTTCTTACAAGAGTCCTGTGGCGAGGACTCAAGTTTCTGCTCCTCCCGGATTTTCTGCTCCCAACAGGTTACCTCCTCCCGGTTTCTCTTCACATCAAAGAGGCGACCTGTCTTCTGATATTGCATCAGGAACTCGTTTACTTGACTCAGCCAATTTGTTGAGGAACGCATATCATGTACCACCTCCAAGTGGAAATTTGAATGCTGCAGGAGATATTGAGTTTATTGATCCTGCAATTTTAGCTGTTGGAAGGGGAAGACTTCATAATGGGATGGAAACAGCAGACTTTGATTTGAGATCAGGGTTCTCTTCTCAACTGAATTCATTTGATAATGATGCAAGACTCCAATTGTTGGCGCAGAGGTCTCTGGCTGCACAACAAGTCAATGGATTTCACGATCCCAGGAACGTAAACAACTTCTCTTCTTCGTTCTCTGACCCATATGGGATTAGCTCGAGGCCAACGGATCAAACACAAGGAACTGGCTTGTCTCCTTTCACACAGCTGCCTAGACAAGCTTCAGCAAATCCATTGTTGTCAAATGGACACTGGGATAATAAGTGGAATGAACCACAAAGTGGTAACAATCTTGGGATTACCCAGCTTCTAAGGAATGAACGGATGGGTTTTAATGACAACGTTTACAGCGGTTTTGAAGAGCCTAAATTCCGGAGGCCCGGTCCTGGAGATCCGTATAACAGAACATACGGGATTGCTAGCatctttctagaagatctcctacaatattctcagctgccatgg

Supplementary File 2D. List of Arabidopsis thaliana plant lines used in this study.

| Plant lines |  |
| --- | --- |
| Transgene or mutant | Genetic background | Line number (if applicable) | Generated via | Vector for floral dip | Reference/ internal seed stock identifier |
| p35S:HA-YFP-NOT9B | Col-0 | 21c | Floral dip | pDS361 | this study/ #PS1704 |
| p35S:HA-YFP-NOT9B | Col-0 | 6d | Floral dip | pDS361 | this study/ #PS1405 |
| p35S:HA-YFP-NOT9B | Col-0 | 24c | Floral dip | pDS361 | this study/ #PS1007 |
| p35S:YFP-NOT9A | Col-0 | 12d | Floral dip | pPS31 | this study/ #PS287 |
| p35S:YFP-NOT9A | Col-0 | 5a | Floral dip | pPS31 | this study/ #PS314 |
| p35S:YFP-NOT9A | Col-0 | 11c | Floral dip | pPS31 | this study/ #PS290 |
| p35S:YFP-NOT9C | Col-0 | 6a | Floral dip | pPS30 | this study/ #PS326 |
| p35S:YFP-NOT9C | Col-0 | 16d | Floral dip | pPS30 | this study/ #PS345 |
| p35S:YFP-NOT9C | Col-0 | 9e | Floral dip | pPS30 | this study/ #PS324 |
| p35S:YFP-NOT9C | Col-0 | 14a | Floral dip | pPS30 | this study/ #PS342 |
| p35S:FLAG-myc-mCherry-NLS-NOT1 M | Col-0 | 5e | Floral dip | pPS191 | this study/ #PS90-5-5 |
| p35S:FLAG-myc-mCherry-NLS-NOT1 M | p35S:HA-YFP-NOT9B 21c | 4a | Floral dip | pPS191 | this study/ #PS98-4-1 |
| p35S:FLAG-myc-mCherry-NLS-NOT1M | p35S:HA-YFP-NOT9B pPHYA:PHYA-CFP #PS524 | 4d | Floral dip | pPS191 | this study/ #PS89-4-4 |
| phyA-211 | Col-0 |  |  |  | NASC 6223/ #PS1707 (Reed et al., 1994) |
| p35S:HA-YFP-NOT9B | phyA-211 |  | Crossing of #PS1704 and #PS1707 |  | this study/ #PS856 |
| pPHYA:PHYA-CFP | phyA-211 |  | Floral dip | #1361 (Genoud et al., 2008) | this study/ #8749 |
| pPHYA:PHYA-NLS-YFP | phyA-211 |  | Floral dip | #2095 | #7129 (Menon et al., 2019) |
| p35S:HA-YFP-NOT9B | pPHYA:PHYA-NLS-YFP |  | Crossing of #PS1704 and #7129 |  | this study/ #PS1831 |
| p35S:HA-YFP-NOT9B | pPHYA:PHYA-CFP |  | Crossing of #PS1704 and #8749 |  | this study/ #PS524 |
| pNOT9:LUC-NOT9B | Col-0 | 12b | Floral dip | pDS398 | this study/ #PS643 |
| pNOT9:LUC-NOT9B | not9b-2 |  | Crossing of not9-2 and #PS643 |  | this study/ #PS1738 |
| not9b-2 | Col-0 |  |  |  | SALKseq_39334/ NASC N925357 (Alonso et al., 2003)/ #PS1882 |
| not9b-1 | Col-0 |  |  |  | SAIL_584_D02/ NASC N824896 (Sessions et al., 2002)/ #PS1441 |
| ago1-27 | Col-0 |  |  |  | (Morel et al., 2002)/#PS1018 |
| p35S:DCP1-CFP | Col-0 | 4a | Floral dip | pPS74 | this study/ #PS2051 |
| p35S:YFP-HA | Col-0 |  | Floral dip | #2969/ pPPO30v1HA (Menon et al., 2019) | this study/ #PS2052 |
| phyB-9 | Col-0 |  |  |  | NASC N6217/ #PS1706 (Reed et al., 1993) |
| p35S:HA-YFP-NOT9B | phyB-9 |  | Crossing of #PS1704 and #PS1706 |  | this study/ #PS983 |
| p35S:HA-YFP-NOT9B (H58A F60A A64Y V71Y) | Col-0 | 7c | Floral dip | pMBag06 | this study/ #PS1444 |
| p35S:HA-YFP-NOT9B (R244E A248L R205D H208D) | Col-0 | 3b | Floral dip | pPS169 | this study/ #PS1227 |
| p35S:HA-YFP-NOT9B (R244E A248L R205D H208D) | Col-0 | 8d | Floral dip | pPS169 | this study/ #PS1306 |
| p35S:NLS-YFP-NOT9B | Col-0 |  | Floral dip | pPS166 | this study/ #PS1350 |
| pPHYA:PHYA-LUC | phyA-211 |  | Floral dip | #2769 | this study/ #9554 |
| p35S:HA-YFP-NOT9B | fhy1-3 fhl-1 |  | Crossing of #PS1704 and #2667/ fhy1-3 fhl-1 (Rösler et al., 2007) |  | this study/ #PS1773 |
| fhy1-3 fhl-1 | Col-0 |  |  |  | #2667/ fhy1-3 fhl-1 (Rösler et al., 2007) |
| Col-0 | n/a |  |  |  | NASC N1092/ #4896 |
| p35S:DCP1-CFP | p35S:HA-YFP-NOT9B | 3c | Floral dip | pPS74 | this study/ #PS2050 |
| pNOT9B:GUS | Col-0 | 4a | Floral dip | pDS396 | this study/ #PS598 |

Supplementary File 2E. Oligonucleotides used for genotyping of mutant lines.

| Line | ID | Genotyping protocol |
| --- | --- | --- |
| not9-1 | SAIL_584_D02/ NASC N824896 | PCR with oPS83 and oPS84 amplifies WT allele, PCR with oPS83 and SAIL-LB3 amplifies not9-1 mutant allele. |
| not9-2 | SALKseq_39334/ NASC N925357 | PCR with oPS278 and oPS279 amplifies WT allele, PCR with oPS278 and SALK-1.3 amplifies not9-2 mutant allele. |
| phyA-211 | NASC N6223 (Reed et al., 1994) | PCR with ah861 and DS543 amplifies WT allele, but not phyA-211. |
| phyB-9 | NASC N6217 (Reed et al., 1993) | PCR with DS941 and DS942; BseLI cuts the phyB-9 amplicon. |

| Name | Sequence (5’→3’) |
| --- | --- |
| ah861 | ACACGATGATTCCTGCATCTG |
| DS543 | GCATATCGCACAAGAGTGTCT |
| DS941 | GGAGCAATGTAGCTAGTGGAAGAAGCTCGACCAGGCTTTG |
| DS942 | CACTAAGTCCATGATACTGGGAC |
| oPS83 | TGGCAGCGAGTTATCAAAGAC |
| oPS84 | CAAGGTTCAGGCACAAGCTAC |
| oPS278 | CCGAACTCAGAGAAAATGCAC |
| oPS279 | ACGGTCTTGATTTGCTTGATG |
| SAIL-LB3 | TAGCATCTGAATTTCATAACCAATCTCGATACAC |
| SALK-1.3 | ATTTTGCCGATTTCGGAAC |

References

Alonso JM, Stepanova AN, Leisse TJ, Kim CJ, Chen H, Shinn P, Stevenson DK, Zimmerman J, Barajas P, Cheuk R, Gadrinab C, Heller C, Jeske A, Koesema E, Meyers CC, Parker H, Prednis L, Ansari Y, Choy N, Deen H, Geralt M, Hazari N, Hom E, Karnes M, Mulholland C, Ndubaku R, Schmidt I, Guzman P, Aguilar-Henonin L, Schmid M, Weigel D, Carter DE, Marchand T, Risseeuw E, Brogden D, Zeko A, Crosby WL, Berry CC, Ecker JR. 2003. Genome-wide insertional mutagenesis of Arabidopsis thaliana. Science (New York, NY) 301:653–657. doi:10.1126/science.1086391

Chapman EJ, Prokhnevsky AI, Gopinath K, Dolja VV, Carrington JC. 2004. Viral RNA silencing suppressors inhibit the microRNA pathway at an intermediate step. Genes Dev 18:1179–1186. doi:10.1101/gad.1201204

Enderle B, Sheerin DJ, Paik I, Kathare PK, Schwenk P, Klose C, Ulbrich MH, Huq E, Hiltbrunner A. 2017. PCH1 and PCHL promote photomorphogenesis in plants by controlling phytochrome B dark reversion. Nat Commun 8:2221. doi:10.1038/s41467-017-02311-8

Genoud T, Schweizer F, Tscheuschler A, Debrieux D, Casal JJ, Schäfer E, Hiltbrunner A, Fankhauser C. 2008. FHY1 mediates nuclear import of the light-activated phytochrome A photoreceptor. PLoS Genet 4:e1000143. doi:10.1371/journal.pgen.1000143

Gibson DG, Young L, Chuang R-Y, Venter JC, Hutchison CA, Smith HO. 2009. Enzymatic assembly of DNA molecules up to several hundred kilobases. Nat Methods 6:343–345. doi:10.1038/nmeth.1318

Hecker A, Wallmeroth N, Peter S, Blatt MR, Harter K, Grefen C. 2015. Binary 2in1 vectors improve in planta (co)localization and dynamic protein interaction studies. Plant Physiol 168:776–787. doi:10.1104/pp.15.00533

Hiltbrunner A, Tscheuschler A, Viczián A, Kunkel T, Kircher S, Schäfer E. 2006. FHY1 and FHL act together to mediate nuclear accumulation of the phytochrome A photoreceptor. Plant Cell Physiol 47:1023–1034. doi:10.1093/pcp/pcj087

Hiltbrunner A, Viczián A, Bury E, Tscheuschler A, Kircher S, Tóth R, Honsberger A, Nagy F, Fankhauser C, Schäfer E. 2005. Nuclear accumulation of the phytochrome A photoreceptor requires FHY1. Curr Biol 15:2125–2130. doi:10.1016/j.cub.2005.10.042

Lampropoulos A, Sutikovic Z, Wenzl C, Maegele I, Lohmann JU, Forner J. 2013. GreenGate - a novel, versatile, and efficient cloning system for plant transgenesis. PLoS One 8:e83043. doi:10.1371/journal.pone.0083043

Menon C, Klose C, Hiltbrunner A. 2019. Arabidopsis FAR-RED ELONGATED HYPOCOTYL 1 and FHY1-LIKE are not required for phytochrome A signal transduction in the nucleus. Plant Comm 1:100007. doi:10.1016/j.xplc.2019.100007

Morel J-B, Godon C, Mourrain P, Béclin C, Boutet S, Feuerbach F, Proux F, Vaucheret H. 2002. Fertile hypomorphic ARGONAUTE (ago1) mutants impaired in post-transcriptional gene silencing and virus resistance. Plant Cell 14:629–639. doi:10.1105/tpc.010358

Rausenberger J, Tscheuschler A, Nordmeier W, Wüst F, Timmer J, Schäfer E, Fleck C, Hiltbrunner A. 2011. Photoconversion and nuclear trafficking cycles determine phytochrome A’s response profile to far-red light. Cell 146:813–825. doi:10.1016/j.cell.2011.07.023

Reed JW, Nagatani A, Elich TD, Fagan M, Chory J. 1994. Phytochrome A and phytochrome B have overlapping but distinct functions in Arabidopsis development. Plant Physiol 104:1139–1149. doi:10.1104/pp.104.4.1139

Reed JW, Nagpal P, Poole DS, Furuya M, Chory J. 1993. Mutations in the gene for the red/far-red light receptor phytochrome B alter cell elongation and physiological responses throughout Arabidopsis development. Plant Cell 5:147–157. doi:10.1105/tpc.5.2.147

Rösler J, Klein I, Zeidler M. 2007. Arabidopsis fhl/fhy1 double mutant reveals a distinct cytoplasmic action of phytochrome A. Proc Natl Acad Sci U S A 104:10737–10742. doi:10.1073/pnas.0703855104

Sessions A, Burke E, Presting G, Aux G, McElver J, Patton D, Dietrich B, Ho P, Bacwaden J, Ko C, Clarke JD, Cotton D, Bullis D, Snell J, Miguel T, Hutchison D, Kimmerly B, Mitzel T, Katagiri F, Glazebrook J, Law M, Goff SA. 2002. A high-throughput Arabidopsis reverse genetics system. Plant Cell 14:2985–2994. doi:10.1105/tpc.004630

Sheerin DJ, Menon C, zur Oven-Krockhaus S, Enderle B, Zhu L, Johnen P, Schleifenbaum F, Stierhof Y-D, Huq E, Hiltbrunner A. 2015. Light-activated phytochrome A and B interact with members of the SPA family to promote photomorphogenesis in Arabidopsis by reorganizing the COP1/SPA complex. Plant Cell 27:189–201. doi:10.1105/tpc.114.134775

Trupkin SA, Debrieux D, Hiltbrunner A, Fankhauser C, Casal JJ. 2007. The serine-rich N-terminal region of Arabidopsis phytochrome A is required for protein stability. Plant Mol Biol 63:669–678. doi:10.1007/s11103-006-9115-x
