# Supplementary material for: Uncovering a novel function of the CCR4-NOT complex in phytochrome A-mediated light signalling in plants
Source: eLife. 2021 Mar 30;10:e63697. doi: 10.7554/eLife.63697 (PMC8009681; doi:10.7554/eLife.63697)
Supplement: Supplementary file 2 [file elife-63697-supp3.odt]

Supplementary File 3. Oligonucleotides for qPCR and antibodies used in this study.

Supplementary File 3A. Oligonucleotides and probes used for qPCR.

| Name | Sequence (5’→3’) |
| --- | --- |
| HY5_fw | ATTTGGAGATCAAAGAAGGAATTGA |
| HY5_rev | CGACAGCTTCTCCTCCAAACTC |
| HY5_probe | FAM-AGCGATGAGGAGATACGGCGAGTGC-TAMRA |
| CHS_fw | CGTGTTGAGCGAGTATGGAAAC |
| CHS_rev | TGACTTCCTCCTCATCTCGTCTAGT |
| CHS_probe | FAM-TCGAGCGCGTGCGTTCTCTTCA-TAMRA |
| NOT9B_pair 1_fw (oPS91) | ATTCTTTGCTCTGGGTCGGG |
| NOT9B_pair 1_rev (oPS92) | TCGCATGCTCTGGGGTTATC |
| NOT9B_pair 2_fw (oPS278) | CCGAACTCAGAGAAAATGCAC |
| NOT9B_pair 2_rev (oPS279) | ACGGTCTTGATTTGCTTGATG |
| ACT1_fw (BE76) | CATCAGGAAGGACTTGTACGG |
| ACT1_rev (BE77) | CATCGACTTGACTCGTCATAC |
| ELIP1_rev (oPS517) | GGAGATGGTGACTTCGGCAA |
| ELIP1_fw (oPS518) | GAGATGCATGGCTGAGGGAG |
| ELIP2_rev (oPS515) | TCCTTGATAGGATCGCCCTGA |
| ELIP2_fw (oPS516) | GCAACGGCGTCGTTTAACAT |
| EPR1_rev (oPS513) | CTCACTTCGATCCTCAGCGG |
| EPR1_fw (oPS514) | TCTGTTTTGTTCGCTTTCAGGC |
| MYBD_fw | TCAAACTCCTGATCCCAACC |
| MYBD_rev.1 | CTCTGTCCATGGAGTTCCTC |
| MYBD_rev.2 | GAAGAAAGGTTATTTTATTAACCTCG |

Supplementary File 3B. List of antibodies used in this study.

| Name | Dilution in PBS-T | Supplier | Host species & clonality | Cat. no. |
| --- | --- | --- | --- | --- |
| αACT | 1:5,000 | Sigma-Aldrich, St. Louis, Missouri | mouse, monoclonal | A0480 |
| αAGO1 | 1:2,000 | Agrisera, Vännäs, Sweden | rabbit, polyclonal | AS09 527 |
| αGFP | 1:1,000 | Sigma-Aldrich, St. Louis, Missouri | mouse, monoclonal | 11814460001 |
| αHA | 1:1,000 | BioLegend, San Diego, California | mouse, monoclonal | 901533 |
| αLUC | 1:2,000 | Sigma-Aldrich, St. Louis, Missouri | mouse, monoclonal | L2164 |
| αmCherry | 1:2,000 | ChromoTek, Planegg, Germany | mouse, monoclonal | 6g6-100 |
| αmouse IgG-AP | 1:7,500 | VectorLaboratories, Burlingame, California | horse, polyclonal | AP-2000-1 |
| αmyc | 1:5,000 | Abcam, Cambridge, UK | mouse, monoclonal | ab32 |
| αPHYA | 1:1,500 | Agrisera, Vännäs, Sweden | rabbit, polyclonal | AS07 220 |
| αrabbit IgG-AP | 1:7,500 | VectorLaboratories, Burlingame, California | goat, polyclonal | AP-1000-1 |
